# Interpreting whole genome sequencing for investigating tuberculosis transmission: a systematic review

**DOI:** 10.1186/s12916-016-0566-x

**Published:** 2016-03-23

**Authors:** Hollie-Ann Hatherell, Caroline Colijn, Helen R. Stagg, Charlotte Jackson, Joanne R. Winter, Ibrahim Abubakar

**Affiliations:** CoMPLEX, University College London, London, WC1E 6BT UK; Centre for Infectious Disease Epidemiology, Infection and Population Health, University College London, London, WC1E 6JB UK; Department of Mathematics, Imperial College London, London, SW7 2AZ UK; Medical Research Council Clinical Trials Unit, 125 Kingsway, London, WC2B 6NH UK

**Keywords:** Whole genome sequencing, Tuberculosis, Transmission, Systematic review

## Abstract

**Background:**

Whole genome sequencing (WGS) is becoming an important part of epidemiological investigations of infectious diseases due to greater resolution and cost reductions compared to traditional typing approaches. Many public health and clinical teams will increasingly use WGS to investigate clusters of potential pathogen transmission, making it crucial to understand the benefits and assumptions of the analytical methods for investigating the data. We aimed to understand how different approaches affect inferences of transmission dynamics and outline limitations of the methods.

**Methods:**

We comprehensively searched electronic databases for studies that presented methods used to interpret WGS data for investigating tuberculosis (TB) transmission. Two authors independently selected studies for inclusion and extracted data. Due to considerable methodological heterogeneity between studies, we present summary data with accompanying narrative synthesis rather than pooled analyses.

**Results:**

Twenty-five studies met our inclusion criteria. Despite the range of interpretation tools, the usefulness of WGS data in understanding TB transmission often depends on the amount of genetic diversity in the setting. Where diversity is small, distinguishing re-infections from relapses may be impossible; interpretation may be aided by the use of epidemiological data, examining minor variants and deep sequencing. Conversely, when within-host diversity is large, due to genetic hitchhiking or co-infection of two dissimilar strains, it is critical to understand how it arose. Greater understanding of microevolution and mixed infection will enhance interpretation of WGS data.

**Conclusions:**

As sequencing studies have sampled more intensely and integrated multiple sources of information, the understanding of TB transmission and diversity has grown, but there is still much to be learnt about the origins of diversity that will affect inferences from these data. Public health teams and researchers should combine epidemiological, clinical and WGS data to strengthen investigations of transmission.

**Electronic supplementary material:**

The online version of this article (doi:10.1186/s12916-016-0566-x) contains supplementary material, which is available to authorized users.

## Background

The ability of whole genome sequencing (WGS) [1] to discriminate between pathogen strains that are indistinguishable using other typing methods has greatly advanced the field of molecular epidemiology. More discrimination is useful for surveillance and outbreak source identification [2], and can lend support to putative transmission events and their direction, particularly for pathogens with little genetic diversity [3]. Despite this advantage, previous reviews of WGS for tuberculosis (TB) [4, 5] and infectious disease in general [1, 6, 7], have highlighted variation in the methods for producing and analysing data leading to heterogeneous results that are difficult to compare. Whilst the capacity to generate WGS data has grown substantially, our understanding of how best to use these data is incomplete.

Although the limited diversity and complicated natural history of TB infection needs special consideration, many of the methods discussed in this review are also employed for studying transmission of other pathogens (e.g. SARS coronavirus [8], methicillin-resistant *Staphylococcus aureus* [9] and *Clostridium difficile* [10]) and many of the issues raised will apply to these pathogens. TB molecular epidemiology using WGS has focussed on four aspects of transmission within outbreaks [5, 6]: identifying chains of transmission; differentiating between relapse and re-infection; measuring within-host diversity and its impact on transmission; and identifying primary versus acquired drug resistance. Awareness of the methods and their limitations should underpin the choice of analytical approaches. This review describes the methods used to analyse WGS data, their limitations and implications for clinical application.

## Methods

The study was conducted, where relevant, in accordance with the Preferred Reporting Items for Systematic Reviews and Meta-Analyses (PRISMA) statement.

### Search strategy and study selection

**Fig. 1:**
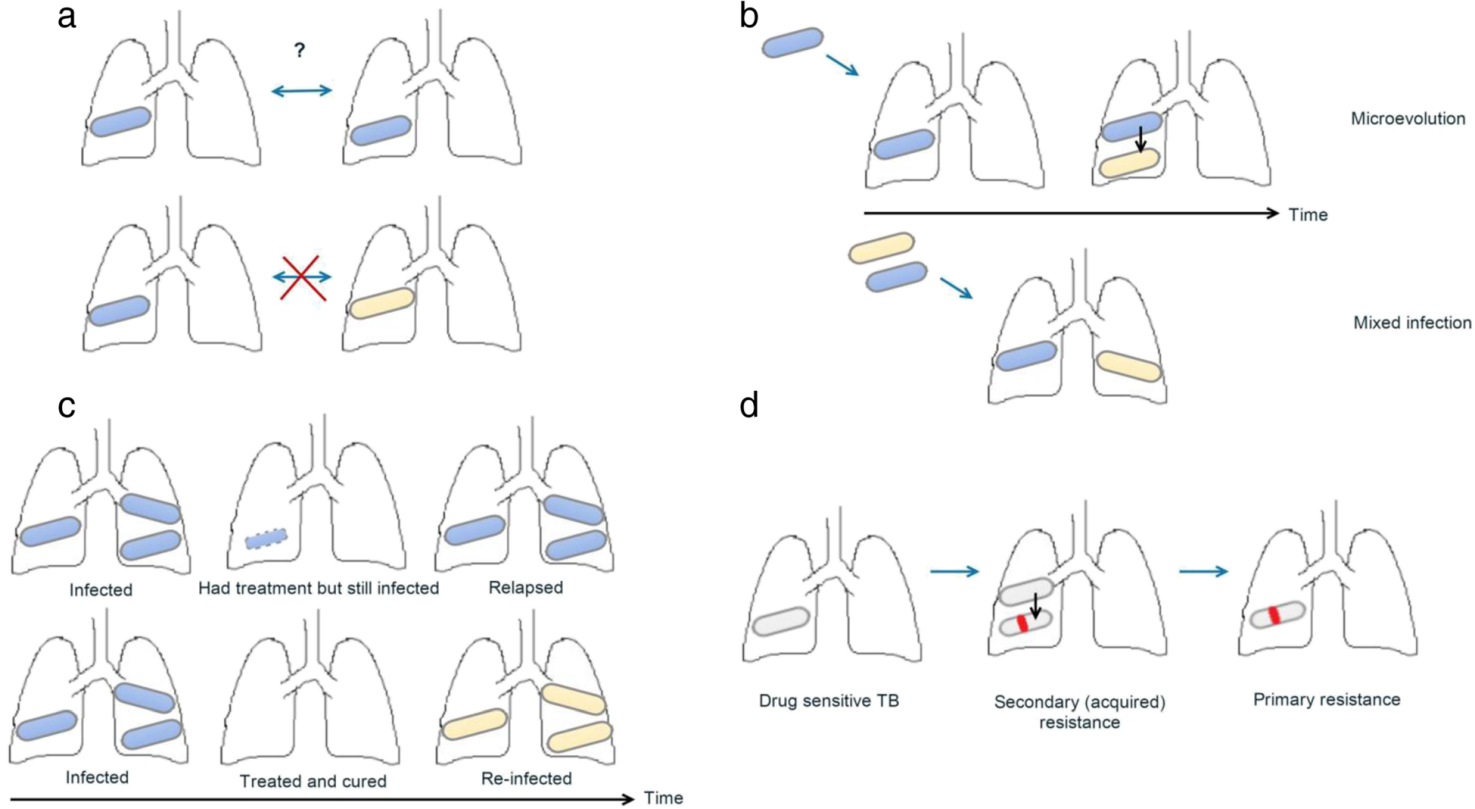
Visual representation of the four topics of the review, with colours representing different strains of TB. **a** Direction of transmission: permissible either way for individuals with the same strain (same colour); excluded for cases with different strains. **b** Within-host diversity, in the first instance as microevolution of an infecting strain and in the second due to mixed infection. A source case with a diverse burden can transmit different combinations of strains. **c** Strain diversity over time. **d** Drug resistance patterns in the form of acquired drug resistance mutations (red line) followed by transmission

Wiley Online Library, ScienceDirect, PubMed, Embase plus Embase Classic, CINAHL, MEDLINE and the Web of Science Core Collection were searched on 14 July 2015 for the key terms and variants of ‘genome sequencing’, ‘tuberculosis’ and ‘transmission’, with no date or language restrictions (see full search strategy in Additional file 1: Appendix A). The reference lists of included articles were also checked for any relevant missing articles. Papers were double-screened by H-AH and JRW and included if they analysed WGS data to investigate the transmission of *Mycobacterium tuberculosis* (*M.tb*), according to any of the four topics prioritised for this review (Fig. 1). Disagreements were resolved by HRS. Reviews, opinion pieces, studies in non-human subjects and of other mycobacteria were excluded.

### Data extraction

Data from each study were extracted by H-AH and HRS independently into a pre-designed spreadsheet that included participant characteristics, the protocol for bioinformatics analysis and the definition of mixed infections, in line with STROME-ID guidelines [11] (Additional file 2: Appendix B). Discrepancies between the reviewers were discussed until consensus was reached.

### Data synthesis and quality assessment

The heterogeneity in methods presented and the results of the included publications rendered meta-analysis inappropriate, thus a narrative synthesis of the main findings is presented. Criteria from STROME-ID and Newcastle-Ottawa were adapted (Additional file 3: Appendix C) to evaluate the molecular and classical epidemiological aspects of study quality as either ‘adequate’, ‘inadequate’ or ‘unknown’. H-AH performed the quality assessment and HRS independently confirmed 10 % of the results. Discrepancies between the reviewers were discussed until consensus was reached.

### Protocol and registration

This review was registered on PROSPERO (CRD42014015633).

## Results

**Fig. 2:**
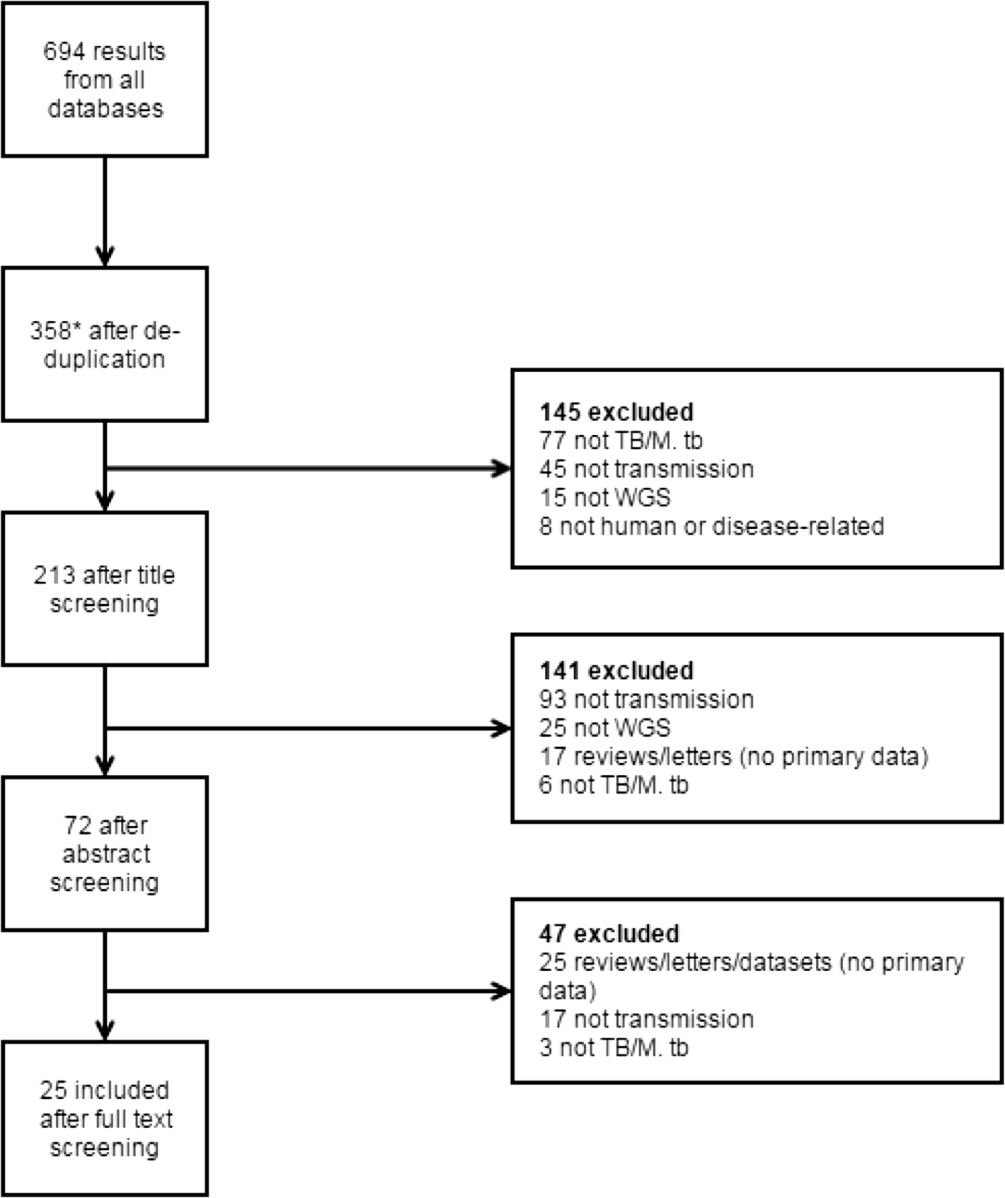
PRISMA flowchart. *Includes one additional study that was found through reference list screening. *M.tb*, *Mycobacterium tuberculosis*; TB, tuberculosis; WGS, whole genome sequencing

Of 358 papers identified after de-duplication (Fig. 2), 25 (reporting on 25 studies) met our inclusion criteria with 97 % inter-reviewer agreement (Additional file 4: Appendix D). Studies investigated one or more of the following: the possibility of transmission regardless of direction (12 studies) [12–23]; the direction of transmission (9 studies) [13, 14, 16, 18, 24–28]; the nature of TB recurrences (4 studies) [18, 24, 29, 30]; within-host strain diversity in the context of transmission (7 studies) [12, 13, 18, 21, 29–31]; and the emergence of drug resistance (6 studies) [23, 32–36]. These studies encompassed a wide range of populations (ages, ethnicities, co-morbidities), countries with varying TB burdens and differing dominant lineage.

### Confirmation of transmission

Twelve studies used WGS to infer transmission (irrespective of direction) by combining information on single nucleotide polymorphisms (SNPs) as a measure of genetic distance, epidemiological data and/or phylogeny (Additional file 4: Appendix D) [12, 14–16, 21, 22]. Identifying clusters and outbreaks reveals the need for public health investigation. Nine studies used a SNP threshold to confirm transmission: Walker et al. [21] investigated SNP differences within community and household clusters in the UK, concluding that a 5 SNP threshold can be used to exclude transmission because they found no epidemiologically linked pairs of isolates exceeding this level of difference. Later studies have similarly defined thresholds using epidemiologically linked or genotypically clustered cases (Table 1) or employed existing SNP thresholds to define transmission clusters [16, 18, 19]. An alternative approach [17] determined the variation between improbable transmission pairs first and, as no pair had less than 2 SNPs difference, used 0–1 SNPs between sequences to define a cluster.

**Table 1:** Studies using SNP thresholds to confirm recent transmission, relapse versus re-infection or microevolution versus mixed infection

Journal article	How was threshold defined?	Cut-off	Sampling fraction	Lineages
Bryant et al. [30]	Own data	≤6 SNPs relapse (same strain); >1,306 re-infection (different)	47 sequenced out of 50 chosen	Four major lineages
Clark et al. [23]	Unknown	<50 SNPs defined a cluster		CAS, LAM, EAI, T1, T2, Beijing, X1
Guerra-Assunção et al. [29]	Own data	≤10 SNPs relapse; >100 re-infection	60 out of 139 WGS confirmed recurrences	Four major lineages
Guerra-Assunção et al. [18]	Own data (transmission); Guerra-Assunção et al. [29] (relapse)	≤10 SNPs confirmed transmission; ≤10 SNPs defined a relapse	1,687 out of 2,332 had WGS	Four major lineages
Kato-Maeda et al. [26]	Own data	0–2 SNPs per transmission event		
Lee et al. [17]	Own data	0–1 SNPs confirmed transmission	631 ‘improbable’ transmission pairs—between outbreak cases and cases in other villages	Outbreak isolates were Euro-American lineage
Luo et al. [16]	Walker et al. [21]			
Roetzer et al. [14]	Own data	3 SNPs confirmed transmission	31 out of 2,301 (for the threshold). Equivalent to eight transmission chains of 2–7 patients	Haarlem lineage
Walker et al. [21]	Own data	≤5 SNPs cluster; >12 SNPs no transmission	303 out of 609 (for the threshold)	All five major lineages
Walker et al. [22]	Own data	475, 1,032 and 1,096 SNPs suggested that patients had been secondarily infected with a different strain rather than within-host evolution	Pulmonary vs extra pulmonary pairs from 49 patients and 110 longitudinal isolates from 30 patients	All five major lineages
Witney et al. [19]	Walker et al. [21]			

Mutation rates were also used to assess whether transmission was likely given the time between samples or how long ago it occurred (assuming that the mutation rate is constant over time) [18, 20]. For example, Guerra-Assunção et al. [18] used a rate of 0.003 SNPs/day with a SNP threshold to exclude links between cases. Others used insertions or deletions to divide clusters into smaller clusters and then precluded transmission between individuals in different clusters [13, 14, 17]. Gardy et al. [12] also split their cluster according to a phylogenetic tree that revealed two lineages and by restricting transmission between the two constructed a transmission network primarily based on contact tracing and timing of infectious periods. In another study, transmission events between epidemiologically linked cases were excluded when the isolates involved were not adjacent on the phylogenetic tree [15].

### Direction

Due to its higher resolution, WGS can reveal variation between isolates that are identical by other typing methods (such as mycobacterial interspersed repetitive units-variable number tandem repeats (MIRU-VNTR)) [37], which may help to infer the direction of transmission between cases. Proposed approaches include SNP accumulation, Bayesian statistical inference and networks. Schürch et al. [24] examined transmission direction using the accumulation of SNPs between isolate sequences from epidemiologically linked patients. The method assumes that over time a strain will acquire new SNPs and retain existing ones, and direction of transmission is to the case with the additional SNPs. This approach has since been applied by others combined with patients’ TB histories and contact tracing data (Table 2) [13, 26, 27] to make it more robust. The studies found small numbers of SNPs amongst small sample sizes (8 SNPs amongst 3 isolates [24], 7 in 9 [26] and 2 in 12 [27]), making this approach easy to implement.

**Table 2:** Methods studies used to confirm direction of transmission

Journal article	How was direction of transmission determined?
Didelot et al. [25]	Epidemiological data and WGS used in a Bayesian inference framework to construct a transmission tree
Gardy et al. [12]	Social network analysis and contact tracing posed putative transmission, timing of infection and smear status was used to narrow down possible direction and WGS to remove transmission events involving cases with different lineages
Kato-Maeda et al. [26]	Contact tracing and accumulation of SNPs
Luo et al. [16]	Epidemiological links and timing of infection and symptoms helped propose direction of transmission between isolates in the same WGS-based cluster. Transmission of mutant alleles from case with mixed base calls
Mehaffy et al. [13]	Genomic and epidemiological information (i.e. SNP pattern, contact information, year of diagnosis and infectiousness based on smear and chest X-ray results)
Pérez-Lago et al. [31]	In one case direction was proposed by the transmission of mutant alleles from a case with mixed base calls
Roetzer et al. [14]	Contact tracing revealed transmission chains and accumulation of variation is mentioned, although not clear if this resolved the order of the chain
Schürch et al. [24]	Accumulation of SNPs
Smit et al. [27]	Accumulation of SNPs and period of infectiousness

Another approach to determine transmission direction is a statistical framework that integrates WGS data with other information to estimate the probabilities of hypothesised transmission chains rather than strictly define transmission events [8, 38]. Didelot et al. used a Bayesian inference method to infer transmission events and their direction from a phylogenetic tree, whilst taking into account within-host diversity [25], and applied it to a TB outbreak of 33 cases in British Columbia, Canada [12]. Such an approach can identify transmission events that a direct analysis from epidemiological and sequence data might not, but quantifying uncertainty in this inference showed that even with WGS, there is considerable uncertainty about transmission events.

Alternatively, studies have used minimum spanning, neighbour joining or median joining networks to visualise transmission using only genomic data [14, 16, 28, 31]. Three studies also created transmission networks but included epidemiological data alongside the genomic data: Walker et al. [21, 22] used their own algorithm to create a similar network, which involved choosing the epidemiological links between cases that had the smallest SNP distance or shortest time; and Schürch et al. [24] used temporal and contact tracing data to assign an index in each SNP cluster and resolve a transmission network.

### Recurrences

Recurrent episodes of TB disease can be classified as relapses or re-infections. The latter implies ongoing transmission, requiring public health action, and suggests a lack of immunity to the newly infecting strain or high intensity of exposure [29, 39], whereas relapse suggests inadequate treatment. To differentiate between relapse and re-infection it is necessary to quantify the genomic differences between isolates from the first and recurrent episodes. There is a fundamental limitation in any genomic investigation of this question because it is possible to be re-infected with a genetically identical strain.

Analyses of data from the REMoxTB trial [30, 40] and the Karonga Prevention Study [29] found a bimodal distribution of pairwise SNP differences between longitudinal isolates: 0–6 [30] or 0–8 SNPs [29] were thought to be relapses; and >1,306 [30] or >100 SNPs [29] re-infections. Both found SNP distances larger than 1,000 when they recovered different lineages from the two episodes. Guerra-Assunção et al. [18] used these results to classify recurrent cases of TB in their Malawian cohort, defining them as relapses if they differed by less than 10 SNPs from the initial strain. In another study, Schürch et al. [24] classified a recurrent case as re-infection because the recurrent strain differed by 1 SNP from the initial infecting strain.

### Within-host diversity

If within-host diversity is not fully captured, transmission might be inappropriately ruled out. For example, if an individual co-infected with two dissimilar strains transmits one of these to a contact, and different strains are then isolated from the two patients, these cases would not be identified as linked [41]. Within-host diversity can arise via mixed infections (a single infection event with multiple distinct strains or repeated infection events with distinct strains i.e. superinfection) or microevolution (within-host evolution).

WGS studies have identified multiple co-infecting TB strains in three ways. First, eight studies considered heterozygous base calls indicative of two strains [18, 19, 21, 23, 26, 28–30]; i.e. if two bases are both likely at a certain position. Definitions of mixed infections in terms of heterozygous base calls have varied (Table 3), and are usually based on the proportion of reads supporting the variant and sometimes the number of mixed base calls across the genome [29, 30]. Heterozygous base calls have also been interpreted as variant subpopulations arising through microevolution [16, 21, 31]. Second, Walker et al. [21] identified a patient as having a mixed infection if two cross-sectional or longitudinal isolates differed by ≥475 SNPs, and conversely, 11 SNPs or less defined microevolution in contrast to the single SNP definition of a re-infection [24]. Third, mixed infections were recognised by one study when an isolate was placed in different lineages of a maximum likelihood phylogenetic tree over multiple constructions [12]. A total of three studies reported mixed infections within their cohort according to their respective WGS definitions: 4 out of 32 isolates [12]; 2 out of 60 pairs of isolates [29]; and 6 out of 47 pairs [30].

**Table 3:** Definitions of heterozygous base calls used to classify mixed infection

Journal article	Mixed infections or microevolution	Definition of heterozygous base call
Bryant et al. [30]	Mixed infection	Mixed base positions were identified at sites where more than one base had been identified in a single sample, where each allele was supported by at least 5 % of reads (minimum read depth of four). Included only positions without strand bias (*p* >0.05), had coverage within the normal range, mapping quality score greater than 50 and base quality scores greater than 30. Sites within 200 base pairs of other heterozygous sites were discounted because of the possibility that they might have been caused by a mapping error. More than 80 heterozygous base calls defined a mixed infection
Guerra-Assunção et al. [18]	Mixed infection	Sample genotypes were called using the majority allele (minimum frequency 75 %) in positions supported by at least 20-fold coverage; otherwise they were classified as missing (thus ignoring heterozygous calls). We excluded samples with >15 % missing genotype calls, to remove possible contaminated or mixed samples or technical errors
Guerra-Assunção et al. [29]	Mixed infection	A position was classified as heterozygous if >1 allele accounted for ≥30 % of the reads (and there were >30 reads). More than 140 heterozygous positions in one sample classified as mixed infection
Kato-Maeda et al. [26]	Mixed infection	Mixed infection was identified when there was a heterozygous base call: 38 % of reads supported the variant; the rest supported reference
Luo et al. [16]	Microevolution	Kept only the calls in which the coverage was ten and the less frequent allele was supported by at least five high-quality reads, as reliable calls. Presence of mixed base calls could indicate microevolution in that patient
Pérez-Lago et al. [31]	Mixed infection	Less frequent nucleotide was supported by five reads
Walker et al. [21]	Microevolution	Suggestive of ‘sub-populations’; i.e. microevolution

Several studies accounted for diversity when investigating transmission. Walker et al. [21] allowed individuals to be part of two (or more) transmission chains by including multiple isolates per person in their networks when it made SNP distances more parsimonious. Similarly, Kato-Maeda et al. [26] considered one isolate which contained a ‘mixed population’ of two other isolates and reflected that one of them may have contained and transmitted the same mixed population but it was not detected. By collecting multiple cross-sectional samples, Pérez-Lago and colleagues [31] were able to build within-host networks and link to other individuals so were better able to resolve the transmission network. Heterozygous base calls have also been used to untangle transmission events: their presence can suggest transmission from a patient with the reference allele followed by microevolution in the second case or microevolution in the first giving rise to an alternative allele followed by transmission to a second case where the alternative allele becomes fixed [16, 31].

Estimates of the within-host mutation rate can be used to better understand transmission: assuming a low mutation rate during latency, one cluster of eight patients with zero SNPs over 9 years was considered evidence of reactivation [13]. However, estimates have differed between studies: using longitudinal data, Walker et al. found the within-host mutation rate to be lower than the mutation rate during household outbreaks (0.3 vs 0.6 SNPs/genome/year) [21]; Guerra-Assunção et al. found the within-host mutation rate higher than between linked pairs in their transmission networks (0.45 vs 0.26 SNPs/genome/year) [18].

Seventeen of the 25 studies reported finding a proportion of isolates recovered from different individuals that were identical, either because there was no diversity or they were unable to capture it. The proportion of identical isolates from the total varied from study to study; Schürch et al. [24] had a cluster of 89 identical isolates out of 104 compared with Luo et al. [16] who found 2 pairs of identical isolates out of 32. The presence of identical isolates makes inference of the direction of transmission impossible based on WGS data alone.

### Drug resistance

WGS studies investigating the emergence of drug resistance have attempted to ascertain whether a resistant strain is being transmitted (primary resistance), requiring more control effort or if resistance is arising separately within individuals (secondary or acquired resistance), suggesting poor drug adherence. Six studies investigated this using similar methods.

Two studies [33, 34] constructed phylogenetic trees and assumed that transmission of a drug-resistant strain had occurred only if all isolates in a cluster had the same resistance-conferring mutation (i.e. the resistance was gained by an ancestor); otherwise drug resistance was considered to have been acquired independently. The studies also used drug resistance-conferring mutations to suggest likely transmission patterns: in one cluster, mutations conferring isoniazid and rifampicin resistance were common amongst all isolates but resistance to fluoroquinolones was not, suggesting that transmission of a multi-drug-resistant (MDR) strain occurred, followed by acquisition of fluoroquinolone resistance in some isolates [33].

Clark et al. [23] used phylogeny and a threshold of 50 SNPs to define potential transmission clusters, but did not require that all isolates within a cluster had the same resistance mutation in order to consider transmission of a resistant strain amongst a proportion of the isolates. With the same principle but a different method, Casali et al. [32] examined 1,000 isolates from Russia and used the number of isolates with a certain resistance mutation in a phylogenetic cluster as a proxy for whether resistance was primary or acquired; i.e. only one case with a certain drug resistance-conferring mutation in a phylogenetic cluster was assumed to represent acquired resistance.

The remaining two studies did not build phylogenetic trees as their isolates were considered to be one outbreak. Ocheretina and colleagues [36] determined that 6 of their 8 isolates had the same resistance mutation for isoniazid and rifampicin, and thus judged that the outbreak represented primary resistance. Regmi et al. [35] employed the same method but only examined 4 of the 54 isolates in their MDR-TB outbreak in Thailand.

### Quality of studies

All 25 studies were assessed for their quality in terms of ten standards (Additional file 3: Appendix C). Inter-reviewer agreement was 86 %. Only a single study was assessed to have an inadequate case definition due to using spoligotyping alone to define their outbreak. Spoligotyping has been shown to have limited discriminatory power compared to 24 loci MIRU-VNTR [42], and thus this study was not comparable to the others included. A single study was determined to be at risk of ascertainment bias due to looking for SNPs in only 8 of 104 outbreak isolates and then establishing whether the other isolates had those specific SNPs only. Given a lack of consensus in the field for defining mixed infections, our assessment considered heterozygous base calls to be adequate and additionally ignored the ill-defined impact of culturing; 64 % of studies did not document mixed infections. Only seven studies (28 %) documented measuring or minimising cross-contamination. The comparison of WGS and epidemiological data was mixed between studies, with a subset (20 %) commenting on epidemiological data but without comparing the number of SNPs separating epidemiologically linked patients.

## Discussion

### Main findings: implications of analytical approaches on WGS inferences

We have identified the range of analytical approaches in using WGS data to infer transmission and its direction, investigate recurrence, describe the impact of within-host variation and assess transmission of resistant strains.

SNP thresholds are common amongst the studies reviewed for defining transmission as well as distinguishing relapse from re-infection [29, 30] and microevolution from mixed infections [21]. They are simple to implement but have limitations. The appropriate value for a SNP threshold is context-specific and will be affected by study-specific factors such as strain diversity in the setting [31, 43], the definition of a quality read, the extent of within-host diversity [15, 22, 25, 31, 38] and the number of amplification steps [1, 44–47] (Additional file 5: Appendix E). Such factors may partially account for apparently conflicting results concerning the SNP differences between linked cases in different studies complicating the comparison of studies: three studies found epidemiological links between cases with larger than 12 SNP differences [15, 22, 31], defined by Walker and colleagues as the upper limit of the SNP distance between epidemiologically linked pairs.

The use of a threshold for transmission relies on finding epidemiologically linked pairs [21]; however, many links may be undetected [48], and the presence or absence of these links does not always prove or disprove transmission. In high incidence settings with endemic strains but no epidemiological links [43], a threshold could suggest transmission incorrectly. Contrarily, unidentified cases may bridge the gap between isolates with large SNP distances, resulting in a mixture of large and small SNP distances for epidemiologically and non-epidemiologically linked isolates [22] that provides no useful cut-off. An alternative to a strict threshold would be to consider the probability of transmission using an approximation to the pairwise distribution of genetic distances [49].

Many studies used the threshold without considering the time between samples, which could erroneously exclude remote transmission events where a large number of SNPs have accumulated. Fundamentally, a threshold relies on a constant mutation rate and despite good agreement for the mutation rate of *M.tb* across multiple epidemiological studies [14, 15, 18, 21], a recent study [15] suggests that the relationship between SNPs and time is affected by resistance and therefore potentially other factors [13] by increasing the mutation rate. This may be because of hitchhiking SNPs (mutations that become fixed because they are physically attached to sites, such as drug resistance genes, that are being selected for [50]) or mutator phenotypes, but there have been many conflicting results [51–56]. The ability of a strain to mutate significantly in a fairly short time would mean a fixed threshold could disregard transmission and classify a relapse as a re-infection or microevolution as mixed infection. Hence it is important to quantify the effect of these factors on the mutation rate, *in vivo* or otherwise.

The within-host mutation rate could similarly be used to investigate the likelihood of relapse versus re-infection and microevolution versus mixed infection. There is uncertainty around whether the mutation rate differs during latency compared to active disease [57, 58]. A lower mutation rate during latency would give rise to less divergence between two cases in a transmission chain with a short latency period, than one with a long latency period. However, the results from the studies reviewed are contradictory [18, 21] and thus more investigation is needed.

**Fig. 3:**
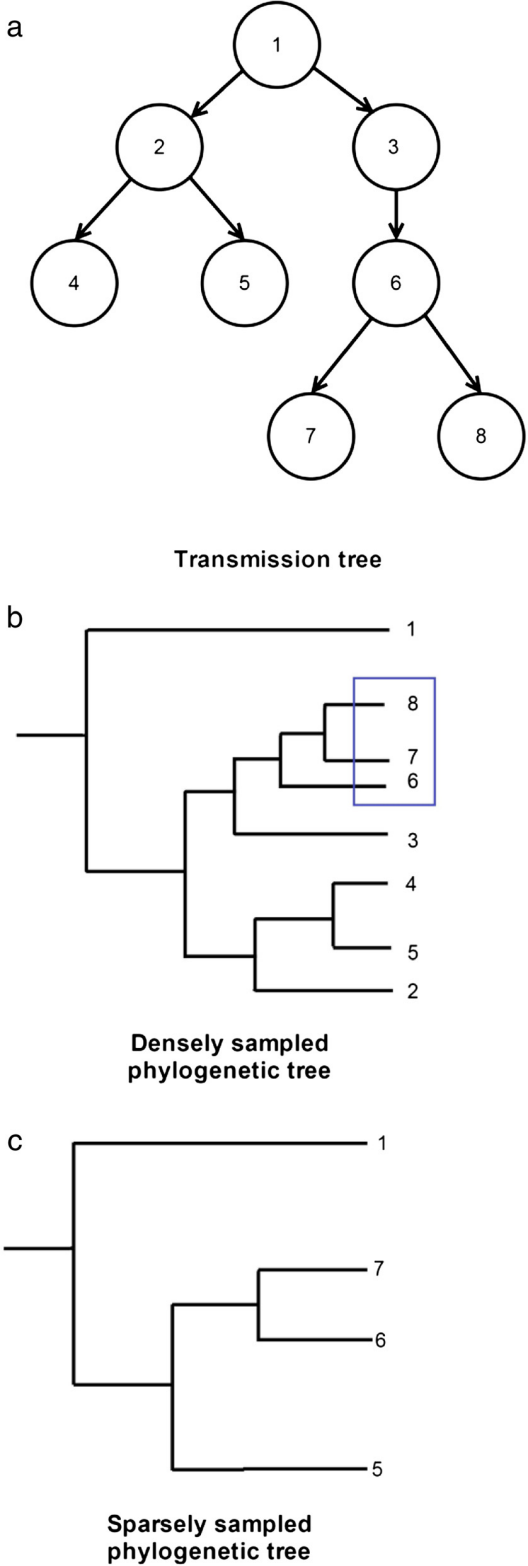
Effect of sampling on the phylogenetic tree. **a** Representation of a transmission tree, where nodes represent individuals, numbers represent the order of infection chronologically and the arrows show the direction of transmission. **b** Phylogenetic tree when all individuals in the outbreak are sampled. Transmission pairs are not necessarily paired on the tree as they may not be the most similar within the context of the outbreak. For example, if we assume that 1 had a long, chronic TB infection then because of the amount of diversity that can accumulate over time it is possible for the genomes from 2 and 3 to be more closely related to each other than to the genome from 1, even though 1 infected them both. This is because the strain that was sampled from 1 has evolved since 1 infected 2 and 3. While rejecting pairs not adjacent on the phylogenetic tree seems sound when sampling is sparse (as transmission pairs would then be relatively rare in the dataset and closer in phylogenetic distance than typical pairs of tips), when sampling is dense (as is desirable in epidemiological investigations). **c** Individuals 2, 3, 4 and 8 have not been sampled for the reconstruction of this tree. This makes the distances between the average pair of tips in the tree larger, highlights the close phylogenetic distance between 6 and 7 and (correctly) suggests transmission occurred between these individuals

Phylogenetic trees have also been used to investigate the possibility of transmission between individuals [15]. Although these trees portray useful information about sequence relatedness, phylogenetic trees are not equivalent to transmission trees [38, 59] and due to their structure, it cannot always be the case that transmission pairs are phylogenetically paired (Fig. 3). Thus excluding transmission on this basis can be misleading. However, phylogenetic trees have been used to resolve transmission in a Bayesian inference framework, which can be useful particularly when manually inspecting SNPs is challenging [25]. This approach assumes dense sampling of cases, which is not often possible without active case finding or with frequent migration. WGS data may leave considerable uncertainty about transmission, which can be mitigated using data such as smear positivity, time since negative tuberculin skin test and individuals’ locations [25].

One of the key gains of WGS in *M.tb* epidemiology is the ability to use SNP accumulation to determine the direction of transmission, as recombination is considered to be absent [60]. Nevertheless, for such a precise method that considers the position and type of each SNP, sequencing errors misinterpreted as SNPs can have a big impact on the inference [61] and homoplasy, although unlikely [62], can be problematic. Inferred direction also depends on the choice of reference genome.

Limited genomic variation, due to the slow *M.tb* mutation rate [13, 27, 63], also hampers methods for determining the direction of transmission. Information on timing of exposure and infectiousness for contacts and cases may help resolve transmission direction [16, 27], although this may conflict with the quantitative interpretation of SNPs [15, 22]. Discordance can be due to ‘casual’ contact resulting in transmission, reactivations from historic infection, poor epidemiological data, or limitations of WGS. Integrating multiple sources of data and allowing for uncertainty in the epidemiological data may allow the best possible understanding of transmission. It is also important to sample within-host diversity thoroughly, but this has practical difficulties. Firstly, a single sputum sample may not contain the full diversity of mycobacteria present in the lungs [64] and may mislead inferences as variants are introduced and purified constantly. A potential solution could be to do longitudinal sampling [4, 65]. Deep sequencing and examination of minor variants can also reveal diversity, and has been attempted in the context of transmission [66]. Secondly, the methods of culturing [67] and obtaining material for sequencing (e.g. selecting single colonies versus sweeping an entire culture plate) can affect the apparent extent of diversity [68, 69].

These difficulties with sampling, and the presence of diversity, may increase the chances of recovering two different strains from individuals linked in transmission. A study by Liu and colleagues presented evidence of multiple strains in an individual’s lung more than 14 SNPs apart that likely occurred due to microevolution after infection [70]. This phenomenon could result in transmission being ruled out if diversity is not detected, particularly if a strict threshold cut-off is used to identify transmission events. However, we are still unable to know how commonly we underestimate diversity, as multiple sampling or sampling directly from lesions is not typically done. Determination of this would make it easier to understand the frequency of undetected diversity, and thus how important it is to be considered by clinicians and researchers.

 Studies looking to differentiate between transmission of drug-resistant strains and acquisition of drug resistance have used similar methods to each other. However, they have differed in whether they needed all isolates within a phylogenetic cluster to share the same resistance-conferring mutation in order to conclude that there was transmission of drug-resistant strains [33, 34]. If they share the same mutation then it is more probable that the mutation arose in an ancestor to the phylogenetic cluster; i.e. the individual earlier in the transmission chain and then the strain was transmitted. However, by assuming this, transmission will be excluded when resistance arose in the middle of a transmission chain and susceptible ancestral isolates are sampled and clustered with the drug-resistant descendants.

Building transmission networks and incorporating resistance mutation data to compare between transmission pairs provides an alternative approach to resolve where resistance is being transmitted versus acquired, aiding interventions.

Several methods are available for examining within-host diversity. Heterozygous base calls have been used to determine microevolution and mixed infections, using WGS; however, a variable and arbitrarily defined threshold number of calls has been used to categorise mixed infection. Incorrectly classifying mixed infections and microevolution can affect inferences about transmission and recurrent disease; better distinction between the two is a topic for future research. With limited diversity, co-infection with strains of the same lineage [29] will not lead to switching between branches of the phylogenetic tree and consequently mixed infections will be missed [12].

### Strengths and limitations

The systematic nature of this review has allowed us to assess available methods for using WGS as a tool for understanding TB epidemiology in detail. However, due to the sometimes small number of studies and the variable approaches to generating sequencing data, quantitative synthesis was not possible. Standard epidemiological quality criteria were often not applicable due to the nature of the investigations.

### Comparison with recent reviews

Recent reviews of WGS for TB have highlighted its use for outbreaks as well as for identifying drug resistance-conferring mutations or reconstructing the evolutionary history of *M.tb* [71, 72]. Kao et al. [1] and Croucher et al. [73] looked at WGS for pathogen outbreak investigations generally, while Takiff et al. [72], Le et al. [74] and Walker et al. [5] reviewed the use of WGS for outbreak investigations of tuberculosis. Our review extends the commentary on the subjects mentioned by these reviews, such as SNP thresholds, relapse versus re-infection and the accumulation of SNPs for determining direction of transmission, and focusses more on the limitations of these methods, as opposed to reviewing the outcomes and their meaning for tuberculosis transmission.

## Conclusions

Applications of WGS for TB have been similar to other infectious diseases; for example, Bayesian inference has been used to infer SARS transmission networks from WGS [8] and the topology of phylogenetic trees has been used to infer outbreaks of *Staphylococcus aureus* [9]. However, because TB is a complex and variable disease, inference of transmission from WGS data for *M.tb* is more difficult. For example, because the latency period is so unpredictable (lasting from weeks to decades) there is uncertainty in ascertaining when an individual was infected and thus the extent to which the infecting strain might have differed from the sampled strain, making inferences of transmission difficult. This is exacerbated by the fact that we have had very little insight into the transmission bottleneck (the number of bacteria transmitted during an infection event), and thus the amount of diversity which may have been transmitted. This has been researched more successfully using WGS for several non-airborne infections (e.g. hepatitis C virus [75]). The need for culturing also provides a barrier to the use of WGS as a rapid public health diagnostic for *M.tb*, as the bacterium is particularly slow-growing.

The WGS studies reviewed here have revealed several findings important for understanding transmission of TB. Diversity plays a significant role in inference of transmission; the finding that there can be large numbers of SNPs between cross-sectional samples from a patient has made it clear that we should be careful when interpreting WGS data. In contrast, many studies have shown that transmission can occur without any diversity arising, which makes it important for us to use other sources of data when trying to build a network of transmission. By using WGS as well as more traditional typing [68], studies have been able to identify superinfection [30], indicating that there may be limited cross-immunity between strains of *M.tb*. There have also been multiple comparisons of MIRU-VNTR and WGS for defining outbreaks. This has revealed that the two markers are not always entirely consistent; for example, there were recorded instances of MIRU-VNTR differences between isolates without SNPs and vice-versa [30].

We have highlighted the limitations and implications of using different approaches for the analysis of WGS data to investigate transmission, and summarise our findings and recommendations in Table 4. Several conclusions can be drawn from this review. Firstly, SNP thresholds have a wide range of applications; however, the genetic distances between sequences should be considered in light of local TB incidence, strain diversity, the time between the samples, potential hitchhiking and homoplasy. Consideration of factors that affect mutation rates is essential. Secondly, epidemiological data and clinical history remain critical to outbreak investigations, especially when sequence data lacks variation. Finally, knowing how diversity arises will help resolve transmission. Better characterisation of microevolution and mixed infection will require better sampling, deeper sequencing and investigation of the within-host mutation rate.

**Table 4:** Findings and recommendations

Over-arching findings from included papers	Recommendations
Suggested SNP thresholds for evidence of transmission are heterogeneous and sensitive to the finding of epidemiological links, SNP calling protocols and culturing/sampling, thus potentially are not transferrable between settings and/or studies	When setting study-specific SNP thresholds consider the time between samples, mutation rate, evolutionary pressure the strain may have been subjected to, and the endemicity of strains. Consider alternative approaches for determining transmission, including Bayesian approaches
The distinction between relapse and re-infection for repeated instances of TB disease has been made empirically (by examining the distribution of SNP distances between the initially infecting and subsequently infecting strains)	While existing thresholds appear adequate for clinical trials, consideration of epidemiological and clinical data is important, as well as a better idea of the within-host mutation rate when more accurate classification is required
The lack of diversity within *M.tb* complicates the use of WGS for inferring transmission patterns (17/25 studies found identical samples). Recent case studies show that there may be more diversity that is not identified by commonly used WGS methods	Deep sequencing, multiple samples and looking at shared minor variants (mutations present at low frequencies) will enhance detection of diversity. Epidemiological data, and consideration of associated uncertainty due to missing contact information, will also be necessary
Examining resistance-conferring mutations shared by phylogenetic clusters is a common method for identifying transmission of drug-resistant strains. However, phylogenetic clusters do not necessarily correspond to transmission clusters	Reconstruction of the transmission tree followed by an examination of the drug resistance patterns between linked individuals may be more appropriate

## Additional files

Additional file 1:
**Appendix A.** Search strategies for databases. (DOCX 28 kb) Additional file 2:
**Appendix B.** Pre-determined data items for extraction. (DOCX 16 kb) Additional file 3:
**Appendix C.** Quality assessment of studies. (DOCX 22 kb) Additional file 4:
**Appendix D.** Included studies and extracted data. (DOCX 32 kb) Additional file 5:
**Appendix E.** Factors affecting the number of polymorphisms detected in sequences. (DOCX 18 kb)

## Abbreviations

*M.tb*: *Mycobacterium tuberculosis*
MDR: multi-drug-resistant
MIRU-VNTR: mycobacterial interspersed repetitive units-variable number tandem repeats
PRISMA: Preferred Reporting Items for Systematic Reviews and Meta-Analyses
SNP: single nucleotide polymorphism
TB: tuberculosis
WGS: whole genome sequencing
