# Supplementary material for: Interpreting whole genome sequencing for investigating tuberculosis transmission: a systematic review
Source: BMC Med. 2016 Mar 23;14:21. doi: 10.1186/s12916-016-0566-x (PMC4804562; doi:10.1186/s12916-016-0566-x)
Supplement: Additional file 1: — Appendix A. Search strategies for databases. (DOCX 28 kb) [file 12916_2016_566_MOESM1_ESM.docx]

**Additional file 1 for ‘Interpreting whole-genome sequencing in investigating tuberculosis transmission: A Systematic Review’**

**Search strategies for each database, the date searched is in brackets**

**Table 1.** Search strategy for MEDLINE (14.07.15)

| Order of search | Search terms | Number of results |
| --- | --- | --- |
| #1 | Tuberculosis[MeSH exploded] or Mycobacterium Tuberculosis[MeSH exploded] or tuberculosis or TB | 230591 |
| #2 | Disease Transmission, Infectious[MeSH exploded] or Disease Outbreaks[MeSH exploded] or Epidemics[MeSH exploded] or epidemiology or Epidemiology[MeSH exploded] or transmi* or outbreak* or pandemic* or spread* or epidemic* or endemic | 926226 |
| #3 | ((whole genome OR full genome OR entire genome OR complete genome OR next generation) ADJ3 sequenc*) OR NGS OR WGS | 22826 |
| #4 | #1 AND #2 AND #3 | 116 |

**Table 2.** Search strategy for EMBASE+classic EMBASE (14.07.15)

| Order of search | Search terms | Number of results |
| --- | --- | --- |
| #1 | Tuberculosis[MeSH exploded] or TB or tuberculosisor Mycobacterium tuberculosis[MeSH exploded] | 317110 |
| #2 | Bacterial transmission[MeSH exploded] or Disease transmission[MeSH exploded] or transmi* or spread* or pandemic* or outbreak* or endemic or epidemic* or Epidemic[MeSH exploded] or Epidemiology[MeSH exploded] or epidemiolog* | 3372564 |
| #3 | ((whole genome OR full genome OR entire genome OR complete genome OR next generation) ADJ3 sequenc*) OR NGS OR WGS | 29589 |
| #4 | #1 AND #2 AND #3 | 160 |

**Table 3.** Search strategy for PubMed (14.07.15)

| Order of search | Search terms | Number of results |
| --- | --- | --- |
| #1 | epidemiolog* or transmi* or spread* or epidemic* or endemic or pandemic* or outbreak* or epidemiology[MeSH terms] or disease transmission, infectious[MeSH terms] or disease outbreaks[MeSH terms] or epidemics[MeSH terms] or pandemics[MeSH terms] | 2511303 |
| #2 | tuberculosis[MeSH Terms] or tuberculosis or TB or mycobacterium tuberculosis[MeSH terms] or mycobacterium tuberculosis | 231531 |
| #3 | ((full genome or complete genome or entire genome or next generation or whole genome) and (sequencing or sequence or sequences)) or NGS or WGS | 101329 |
| #4 | #1 AND #2 AND #3 | 197 |

**Table 4.** Search strategy for Web of Science Core collection (14.07.15)

| Order of search | Search terms | Number of results |
| --- | --- | --- |
| #1 | Epidemiolog* or outbreak* or transmi* or pandemic* or epidemic* or endemic | 1489972 |
| #2 | Tuberculosis or TB or “mycobacterium tuberculosis” | 151403 |
| #3 | ((“full genome” OR “whole genome” OR “complete genome” OR “entire genome” OR “next generation”) NEAR/3 sequenc*) OR NGS OR WGS | 30603 |
| #4 | #1 AND #2 AND #3 | 184 |

**Table 5.** Search strategy for CINAHL (14.07.15):

| Order of search | Search terms | Number of results |
| --- | --- | --- |
| #1 | Disease Transmission[MeSH exploded] or Disease Outbreaks[MeSH] or Epidemiology[MeSH exploded] or transmi* or epidemiolog* or spread* or outbreak* or epidemic* or endemic | 632323 |
| #2 | Tuberculosis[MeSH exploded] or Mycobacterium tuberculosis[MeSH] or TB or tuberculosis | 17827 |
| #3 | ((full genome or complete genome or whole genome or entire genome or next generation) N3 sequenc*) OR NGS OR WGS | 720 |
| #4 | #1 AND #2 AND #3 | 12 |

**Table 6.** Search strategy for ScienceDirect (14.07.15):

| Order of search | Search terms | Number of results |
| --- | --- | --- |
| #1 | ((“full genome” or “ whole genome” or “complete genome” or “entire genome” or “next generation”) W/3 sequenc*) or WGS or NGS | 893820 |
| #2 | Tuberculosis or TB or “mycobacterium tuberculosis” | 189310 |
| #3 | Epidemiolog* or outbreak* or spread* or pandemic* or epidemic* or transmi* or endemic | 3244319 |
| #4 | TITLE-ABSTR-KEY(#1 AND #2 AND #3) | 16 |

**Table 7.** Search strategy for WILEY (14.07.15)

| Order of search | Search terms | Number of results |
| --- | --- | --- |
| #1 | ((whole genome OR full genome OR entire genome OR complete genome OR next generation) AND sequenc*) OR WGS OR NGS | 311853 |
| #2 | Tuberculosis or tb or “mycobacterium tuberculosis” | 221222 |
| #3 | Epidemiolog* or transmi* or spread* or epidemic* or pandemic* or outbreak* or endemic | 1770174 |
| #4 | Abstract(#1 AND #2 AND #3) | 9 |
