# Supplementary material for: Interpreting whole genome sequencing for investigating tuberculosis transmission: a systematic review
Source: BMC Med. 2016 Mar 23;14:21. doi: 10.1186/s12916-016-0566-x (PMC4804562; doi:10.1186/s12916-016-0566-x)
Supplement: Additional file 2: — Appendix B. Pre-determined data items for extraction. (DOCX 16 kb) [file 12916_2016_566_MOESM2_ESM.docx]

**Additional file 2 for ‘Interpreting whole-genome sequencing in investigating tuberculosis transmission: A Systematic Review’**

**Data items for extraction**

**Table 1.** Predetermined data for extraction

| General | Bioinformatics | Phylogenetic tree | Mixed infections | Relapses | Direction | Limitations |
| --- | --- | --- | --- | --- | --- | --- |
| Aim | % of the genome covered by reads | Method (maximum likelihood, Bayesian etc.) | How were mixed infections defined | How were relapses defined? | How was direction determined? | Small sample |
| Theme | Reference genome | Software | What was the effect on transmission? |  |  | Culturing method |
| Number of individuals in study | Software | How were SNPs used |  |  |  | Missing isolates |
| How were the samples identified | Sequencing machine |  |  |  |  | Other |
| Country | Other information |  |  |  |  |  |
| Incidence rate classification (High ≥40 cases per 100,000, Low <40 cases per 100,000) |  |  |  |  |  |  |
| Type of study |  |  |  |  |  |  |
| Sample type |  |  |  |  |  |  |
| Was epidemiological/contact tracing data used |  |  |  |  |  |  |
| Population type (convenience, representative, epidemiologically/genotypically clustered, other) |  |  |  |  |  |  |
| Exclusion criteria |  |  |  |  |  |  |
| When and how were isolates collected |  |  |  |  |  |  |
| Length of period of collection |  |  |  |  |  |  |
| How long was follow-up (for recurrent disease) |  |  |  |  |  |  |
