# Supplementary material for: Interpreting whole genome sequencing for investigating tuberculosis transmission: a systematic review
Source: BMC Med. 2016 Mar 23;14:21. doi: 10.1186/s12916-016-0566-x (PMC4804562; doi:10.1186/s12916-016-0566-x)
Supplement: Additional file 3: — Appendix C. Quality assessment of studies. (DOCX 22 kb) [file 12916_2016_566_MOESM3_ESM.docx]

**Additional file 3 for ‘Interpreting whole-genome sequencing in investigating tuberculosis transmission: A Systematic Review’**

**Quality assessment**

**Table 1: Quality assessment of studies**

|  | Was the infectious-disease case definition appropriate? Did they used appropriate diagnosis methods? | Were measures taken to minimise and measure cross-contamination? | Was the timeframe of the study appropriate? (3 years minimum set as a threshold where transmission examined) | Were the participants representative? | If the study investigates molecular clusters, did they state the sampling fraction? | Were methods used to detect multiple-strain infections appropriate? Was their effect on the study findings included? | Were efforts made to address discovery or ascertainment bias? | Did the study consider alternative explanations for findings when transmission chains are being investigated, and report the consistency between molecular and epidemiological evidence? | Was follow-up time long enough for outcomes to occur? (≥1year) | Was sample size justified, where a number was decided before the study was undertaken? (for hypothesis driven studies) |
| --- | --- | --- | --- | --- | --- | --- | --- | --- | --- | --- |
| Bryant *et al*. (BMC Infectious Diseases, 2013) | Adequate | Unknown | Adequate | Adequate | Inadequate | Unknown | Adequate | Adequate/Adequate | N/A | N/A |
| Bryant *et al.* (The Lancet Resp Med, 2013) | Adequate | Adequate | N/A | Unknown | N/A | Adequate/Adequate | Adequate | N/A | Adequate | N/A |
| Casali *et al.* | Adequate | Unknown | Inadequate | Adequate | N/A | Unknown | Adequate | N/A/Adequate | N/A | N/A |
| Clark *et al.* | Adequate | Adequate | Adequate | Unknown | Adequate | Adequate/Inadequate | Adequate | N/A/Adequate | N/A | N/A |
| Didelot *et al.* | Unknown | Unknown | Unknown | Unknown | Unknown | Unknown | Unknown | Adequate/Adequate | N/A | N/A |
| Gardy *et al.* | Adequate | Unknown | Adequate | Adequate | Adequate | Inadequate/Inadequate | Adequate | Adequate/Inadequate | N/A | N/A |
| Guerra-Assuncao *et al*. (2015) | Adequate | Adequate | Adequate | Adequate | Adequate | Adequate/N/A | Adequate | Adequate/Adequate | Adequate | N/A |
| Guerra-Assuncao *et al.*(2014) | Adequate | Adequate | N/A | Adequate | N/A | Adequate/Adequate | Adequate | N/A | Adequate | N/A |
| Ioerger *et al.* | Adequate | Unknown | Unknown | Unknown | Inadequate | Unknown | Adequate | N/A | N/A | N/A |
| Kato-Maeda *et al.* | Adequate | Unknown | Inadequate | Adequate | Adequate | Adequate/Adequate | Adequate | Adequate/Adequate | N/A | N/A |
| Lanzas *et al.* | Adequate | Adequate | Adequate | Unknown | N/A | Unknown | Adequate | N/A | N/A | N/A |
| Lee *et al.* | Adequate | Adequate | Adequate | Adequate | Adequate | Unknown | Adequate | Inadequate/Adequate | N/A | N/A |
| Luo *et al*. | Adequate | Unknown | Inadequate | Adequate | Adequate | Unknown | Adequate | Inadequate/Adequate | N/A | N/A |
| Martin Williams *et al.* | Adequate | Unknown | Adequate | N/A | N/A | Unknown | Adequate | Inadequate/Adequate | N/A | N/A |
| Mehaffy *et al.* | Adequate | Unknown | Adequate | Adequate | Adequate | Adequate/N/A | Adequate | Inadequate/Inadequate | N/A | N/A |
| Ocheretina *et al.* | Inadequate | Unknown | Adequate | Adequate | Adequate | Unknown | Adequate | Inadequate/Adequate | N/A | N/A |
| Perez-Lago *et al.* | Adequate | Unknown | Adequate | Adequate | Adequate | Unknown | Adequate | Inadequate/Adequate | N/A | N/A |
| Regmi *et al.* | Adequate | Unknown | Adequate | Inadequate | Adequate | Unknown | Adequate | N/A | N/A | N/A |
| Roetzer *et al.* | Adequate | Unknown | Adequate | Adequate | Adequate | Unknown | Adequate | Inadequate/Inadequate | N/A | N/A |
| Schurch *et al.* | Adequate | Unknown | Adequate | Adequate | Adequate | Unknown | Inadequate | Adequate/Inadequate | Adequate | N/A |
| Smit *et al.* | Adequate | Unknown | Inadequate | Adequate | Adequate | Unknown | Adequate | Inadequate/Adequate | N/A | N/A |
| Stucki *et al.* | Adequate | Unknown | Adequate | Adequate | Adequate | Adequate/Inadequate | Adequate | Inadequate/Inadequate | N/A | N/A |
| Walker *et al.* (2014) | Adequate | Adequate | Adequate | Adequate | Adequate | Unknown | Adequate | Inadequate/Adequate | N/A | N/A |
| Walker *et al.*(2013) | Adequate | Unknown | Adequate | Adequate for clusters/Unknown for cross-sectional/longitudinal patients | Inadequate | Unknown | Adequate | Inadequate/Adequate | N/A | N/A |
| Witney *et al.* | Adequate | Unknown | Adequate | Adequate | N/A | Adequate/N/A | Adequate | N/A/Adequate | N/A | N/A |

Unknown = not mentioned, Adequate = considered to not be at risk of bias, Inadequate = considered to be at risk of bias, N/A = due to the nature of the study this is not able to be considered
