# Supplementary material for: Interpreting whole genome sequencing for investigating tuberculosis transmission: a systematic review
Source: BMC Med. 2016 Mar 23;14:21. doi: 10.1186/s12916-016-0566-x (PMC4804562; doi:10.1186/s12916-016-0566-x)
Supplement: Additional file 4: — Appendix D. Included studies and extracted data. (DOCX 32 kb) [file 12916_2016_566_MOESM4_ESM.docx]

| **Additional file 4 for ‘Interpreting whole-genome sequencing in investigating tuberculosis transmission: A Systematic Review’**  **Table 1. Summary of included studies** | | | | | | | | | | | | |  | |  | |
| --- | --- | --- | --- | --- | --- | --- | --- | --- | --- | --- | --- | --- | --- | --- | --- | --- |
| **Journal article** | **Participants** | **Country (TB burden*)** | **Sample size** | **Type of study** | **Length of study** | **Focus** | **Sequencing machine** | **Reference genome** | **Patient characteristics** | **Lineages** | **Quality of SNP** | **Read length** | | **Max no. of SNPs** | |  |
| Bryant *et al*.(2013, BMC Infectious Diseases) | RFLP clusters with epidemiological links | Netherlands (Low) | 199 | Retrospective | - | Confirmation | Illumina Genome Analyzer IIx | H37Rv | Drug resistant | Four Global lineages (Euro-American, East-African Indian, East Asian, Indo-Oceanic) | Alleles need support of $\geq$75% of reads on each strand, base quality score ≥ 50 and mapping quality score ≥30.  Repetitive regions are avoided. | 76/108 bp | | Pairwise SNP distances: range 0-149, mean 3.42.  11,879 variable positions found over all isolates | |  |
| Bryant *et al.*(2013, The Lancet Resp Med.) | RCT participants: previously untreated, drug-sensitive, smear-positive pulmonary TB without severe co-morbidities | Malaysia, South Africa and Thailand (High) | 47 pairs | Retrospective observational | Patients were observed for 18 months, including treatment and follow-up | Recurrences and Diversity | Illumina HiSeq | H37Rv | No severe co-morbidities. Drug sensitive | Four Global lineages (Euro-American, East-African Indian, East Asian, Indo-Oceanic) | SNPs in the PE and PPE genes that differed between the relapse pairs were discounted. SNP quality as above. | 100 bp | | Pairwise SNP distances: range 0-1419, mean 113.278 (relapse/re-infection pairs). 10,354 variable positions. | |  |
| Casali *et al.* | Representative sample of patients with pulmonary disease. Culture-proven. | Russia (High) | 1000 | Prospective | 2 years | Resistance | Illumina Genome Analyzer IIx or HiSeq 2000 | H37Rv | Drug resistance (MDR and XDR) | Beijing, Central Asian Strain, Euro-American and East-African Indian | Alleles need support of >70% of reads, including ≥ 5 in each direction and mapping quality ≥ 45. Repetitive regions were avoided. | 54/75/ 100 bp | | SNP distances between linked cases: range: 0-183 SNPs.  32,445 variable positions | |  |
| Clark *et al.* | Convenience sample of treatment experienced TB patients (69% of MDR-TB cases in Uganda) | Uganda (High) | 51 samples (41 patients) |  | 4 years | Resistance and Confirmation | Illumina HiSeq 2000 | H37Rv | HIV present. Age: 19-50, Males and females. MDR | Central Asian Strain, Beijing, East-African Indian | Only variants of high quality ($\geq$Q30) and supported by bi-directional reads were retained. Variants in PPE/PE loci were excluded. | 76 bp | | Range 0-1060 (compared to reference). 6857 variable positions in total. SNP distances between linked cases: 0-32 | |  |
| Didelot *et al.* | Outbreak cases, defined by the same MIRU-VNTR and contact tracing | Canada (Low) | 33 |  |  | Direction | Illumina HiSeq | CDC1551 | - | - | Retained positions called with quality score of 222, genotype quality of 99, and no indication of strand basis or low depth of coverage. SNV excluded if located within 50bp of another SNV. |  | |  | |  |
| Gardy *et al.* | Outbreak cases, defined by the same MIRU-VNTR and contact tracing | Canada (Low) | 32 sequenced | Retrospective | 2 years | Confirmation and Diversity | Illumina Genome Analyzer II | CDC1551 | Age: 1-71, Males and Females | - | Excluded: i) SNPs with quality scores <30; ii) SNPs occurring in clusters (i.e. within 10 bp of each other); iii) SNPs identical across all 36 isolates; and iv) 15 SNP positions at which one or more isolates displayed an ambiguous residue call | 50 bp | | 204 SNPs amongst all isolates | |  |
| Guerra-Assunção *et al.* (2015) | Culture confirmed cases in Karonga district | Malawi (High) | 1687 sequenced with high quality data |  | 15 years | Diversity, Direction, Recurrences and confirmation | Illumina HiSeq 2000 | H37Rv | Age: <20-50+. HIV present. Males and females | East Asian, Euro American, Indo-Oceanic, East-African Indian | Removed low-quality sequences and low-quality 3′ ends of reads, retaining only reads ≥ 50 bp long, with nucleotides above quality score Q27. Excluded samples with coverage less than 10-fold or with >15% missing genotypes. Excluded genome positions with >15% missing genotypes and those in highly repetitive regions. | 100 bp | | Paired SNP distances: 0-almost 2000 | |  |
| Guerra-Assunção *et al.* (2014) | Laboratory confirmed TB cases who had completed treatment | Malawi (High) | 60 pairs with WGS | Population-based | 14 years | Recurrences and Diversity | Illumina HiSeq 2000 | H37Rv | HIV present. Age: <30 – 50+, Males and Females | East Asian, Euro American, Indo-Oceanic, East-African Indian | Removed low-quality sequences and low-quality 3′ ends of reads, retaining only reads ≥ 50 bp long, with nucleotides above quality score Q27. Excluded SNPs with >15% missing genotypes and those in highly repetitive regions. | 100 bp | | Paired SNP distances: 0-1000+ | |  |
| Ioerger *et al.* | Two RFLP drug resistant clusters | South Africa (High) | 14 |  |  | Resistance | Illumina Genome Analyzer II | H37Rv/HN878 | Drug resistance. | Beijing |  | 36 bp | | 1546 SNPs in sample | |  |
| Kato-Maeda *et al.* | Individuals found through contact tracing to be involved in a transmission chain | USA (Low) | 9 | Population-based | 22 months | Direction | Illumina Genome Analyzer | H37Rv | HIV absent. Hispanic males. Age: 18 – 34. Drug susceptible. | - | SNPs in PE, PE-PGRS, PPE genes and mobile elements were excluded. 25 putative SNPs, ($\geq$85% of reads supported one base call and ≥ 12 reads depth), were analyzed with PCR Sanger method. 7 confirmed as true SNPs. |  | | 7 SNPs between all isolates | |  |
| Lanzas *et al.* | 66 MDR and 31 drug sensitive patients | Panama (High) | 97 |  | 10 years | Resistance | Illumina Genome Analyzer IIx | H37Rv | HIV present. Age: 14 – 81. Males and females. MDR and drug susceptible. | Mainly Latin American-Mediterranean | Needed depth of coverage $\geq$25% of the mean, and the majority nucleotide represented in >70% of reads; gaps and regions with clusters of SNPs were excluded. | 36-54 bp | | 6,890 variable positions | |  |
| Lee *et al.* | Outbreak cases | Canada (Low) | 78 sequenced (out of 82) |  | 22 years | Confirmation | Illumina MiSeq 250 | H37Rv |  | Euro-American | Excluded SNPs with Phred score <50 | 50+ bp | |  | |  |
| Luo *et al.* | Two clusters based on MIRU-VNTR and SNP typing | China (High) | 32 sequenced | Population-based | 1 year | Confirmation and Direction | Illumina HiSeq | H37Rv | Age: 17 – 79. Males and females. MDR and non-MDR. | Beijing | SNPs with coverage <3 and SNPs in the PE/PPE, PE-PGRS and drug-resistance associated genes were filtered | 300 bp | | SNP distances for linked cases: 0-100+ | |  |
| Martin Williams *et al.* | Patients with identical MIRU-VNTR to first identified case | UK (Low) | 4 (plus outbreak strain and 36 South Africa strains for comparison) |  |  | Confirmation | Illumina MiSeq | H37Rv |  |  |  |  | |  | |  |
| Mehaffy *et al.* | Cluster based on spoligotyping and MIRU-VNTR | Canada (Low) | 56 isolates (53 patients) |  | 17 years | Direction, Diversity and Confirmation | Illumina | H37Rv | Age: 20 – 74.  Males and females. HIV present. All drug susceptible. |  | SNPs required a minimum read depth of 20X and a variant frequency of at least 75. SNPs  in the PE, PPE and PE_PGRS gene were excluded. |  | | 722 SNPs compared to H37Rv | |  |
| Ocheretina *et al.* | Isolates sharing the same drug-resistance mutation | Haiti (High) | 7 sequenced |  | 5 years | Resistance | Illumina HiSeq 2000 | H37Rv |  |  | Excluded SNPs in PPE, PE-PGRS and wag22 genes and where one or more isolates displayed an ambiguous residue with over 20% match with reference alleles | 50 bp | | 755 variant positions compared to H37Rv, 22 SNPs and 1 deletion between 6 isolates | |  |
| Pérez-Lago *et al.* | Epidemiologically supported MIRU-VNTR and RFLP clusters with at least one clonal variant | Spain (Low) | 36 |  | 7 years | Diversity and Direction | Illumina HiSeq | MRCA of the MTBC | - | Euro-American | SNP calls of low quality: minimum coverage 10, minimum mapping quality of the SNP 20 | 51-101 bp | | Within cluster SNP distances: 0-18 | |  |
| Regmi *et al.* | Cluster define by MIRU-VNTR and spoligotyping | Thailand (High) | 4 isolates sequenced (54 total) |  | 6 years | Resistance | Illumina HiSeq 2000 | H37Rv | - | Beijing | Phred quality  score of ≤20 and SNVs with coverage of fewer than 10  reads were discarded. Additionally, heterozygous SNVs  with allele frequencies of <75 % that were commonly present  in all four isolates were discarded | 100 bp | | 1242 common SNPs between outbreak isolates and reference | |  |
| Roetzer *et al.* | Large strain cluster (Haarlem lineage), identified by RFLP and MIRU-VNTR | Germany (Low) | 86 | Prospective population-based | 14 years | Confirmation and Direction | Illumina | H37Rv | HIV present. Age: 2 – 83. Males and females. Drug susceptible. | Haarlem | SNPs needed a minimum coverage of 10 reads and a minimum allele frequency of 80% as thresholds for detection. |  | | 85 SNPs in sample. SNP distances between linked cases: 0-3 | |  |
| Schürch *et al.* | Harlingen cluster (RFLP with contact tracing) | Netherlands (Low) | 3 sequenced (104 checked for 8 SNPs) |  | 16 years | Direction and Recurrences | GS FLX Titanium |  | - | - | 8 polymorphic SNPs were verified by subsequent resequencing on an ABI 3730xl sequencer | 400 bp | | 8 SNPs between 3 isolates | |  |
| Smit *et al.* | Clustered with spoligotype and MIRU-VNTR | Finland (Low) | 12 outbreak + 7 historical sequenced (14 in total) |  | 1 year | Direction | - | - | Age: 16-23 years | - | Single-nucleotide polymorphisms (SNPs) were  considered valid if supported by at least two and .70% of mapped reads on each strand with a minimum  mapping quality of 45 |  | |  | |  |
| Stucki *et al.* | Cluster isolates identified with SNP typing | Switzerland (Low) | 69 isolates sequenced |  | 20 years | Direction | Illumina | Inferred common ancestor of all MTBC lineages | Age: 34-53 years.  HIV present.  Males and females. | - | SNPs with a coverage of ≥10  reads and Phred-score≥20.  SNPs in “PE/PPE/PGRS,” “maturase,”  “phage,” “insertion sequence,” or “13E12 repeat family protein”  genes or with missing nucleotide  calls in at least 3 isolates were excluded. The short-read alignment tool SMALT was also used to call SNPs.  Only positions  called by both after filtering  were included. | - | | 133 variable positions amongst the 69 isolates | |  |
| Walker *et al.* (2013) | Random cross-sectional and longitudinal isolates from single patients. Isolates from community MIRU-VNTR and household clusters | UK (Low) | 390 isolates (254 patients) | Retrospective observational | Archived between 1994 and 2011 | Confirmation and Diversity | Illumina HiSeq | H37Rv | - | Beijing, European American, Central Asian, East-African Indian | >75% of reads needed to support variant calls, which had to be homozygous in a diploid model. Only variants supported by ≥5 reads, including one in each direction that did not occur at sites with unusual depth and were not within 12 bp of another nucleotide variant, were accepted. | 75 bp | | Pairwise SNP distances: 0-5 for linked cases, 0-150 for unlinked cases  1,096 SNPs was the largest pairwise distance between longitudinal isolates | |  |
| Walker *et al.*(2014) | Unselected, geographically restricted population | UK (Low) | 247 | Observational | 6 years | Confirmation | Illumina HiSeq | H37Rv | Age: 1-89 | - | Variant calls in non-repetitive regions were made providing they were supported by $\geq$5 reads, including one in each direction. Sites where minority variants represented >10% of read depth were defined as mixed and no base called. |  | | SNP distances between linked cases: 0-7 (median 1)  Median pairwise SNP distances 1106 (857-1715) without secondary cases from each genomic cluster | |  |
| Witney *et al.* | Six hospital patients with suspected XDR-TB | UK (Low) | 16 isolates (6 patients) |  | 7 years | Confirmation | Ion Torrent personal genome machine | H37Rv | - | Beijing | mapping quality of >30, site quality score of >30,  $\geq$4 reads covering each site with $\geq$2 reads mapping to each strand but  with a maximum depth of coverage of 200x, $\geq$75% of reads supporting  the site, and an allelic frequency of 1. |  | | 33-297 pairwise SNP distances | |  |

bp = base pairs, MRCA = Most recent common ancestor, MTBC = *M. tb* complex. *TB burdens of countries were taken from Public Health England[^26^](#_ENREF_26) with high burden defined as >40 cases/100,000
