# Supplementary material for: Interpreting whole genome sequencing for investigating tuberculosis transmission: a systematic review
Source: BMC Med. 2016 Mar 23;14:21. doi: 10.1186/s12916-016-0566-x (PMC4804562; doi:10.1186/s12916-016-0566-x)
Supplement: Additional file 5: — Appendix E. Factors affecting the number of polymorphisms detected in sequences. (DOCX 18 kb) [file 12916_2016_566_MOESM5_ESM.docx]

**Additional file 5 for ‘Interpreting whole-genome sequencing in investigating tuberculosis transmission: A Systematic Review’**

**Table 1: The effect of study specific factors on the number of polymorphisms detected in sequences**

| **Study factors** | **Effect on number of SNPs detected** |
| --- | --- |
| Study duration | Assuming that mutations occur and become fixed as time evolves, the longer the duration of study the more polymorphisms that will have occurred and been fixed in the population (so this affects the number of SNPs found in the study overall not between related cases) |
| Strain diversity | If there are highly diverse strains in the population then large SNP distances will be found between pairs of sequences |
| Sequencing machine | Sequencing machines (e.g. Illumina) require the sample to be cultured before it is sequenced. This can reduce the number of polymorphisms detected by causing a bottleneck |
| Length of reads | The longer the read, the more SNPs found[^1^](#_ENREF_1) |
| Coverage | The deeper the coverage, the more polymorphisms likely to be found |
| Definition of quality read/SNP | The definition of a quality read will affect the number of SNPs ‘confirmed’ as the definition relies on support from a certain number of reads. Thus more stringent rules on quality reads will mean fewer reads to support variants. Stringent definitions of SNPs requiring high confidence in variants will result in fewer SNPs found. |
| Bioinformatics software | Factors such as the internal filtering criteria may affect the number of polymorphisms found[^2^](#_ENREF_2) |
| Number of amplification steps | The more amplification steps, the more errors are likely to be introduced[^3^](#_ENREF_3) resulting in polymorphisms |
